# Laboratory Features of Trichinellosis and Eosinophilia Threshold for Testing, Nunavik, Quebec, Canada, 2009–2019

**DOI:** 10.3201/eid2812.221144

**Published:** 2022-12

**Authors:** Luke B. Harrison, Michael D. Libman, Chelsea Caya, Momar Ndao, Cedric P. Yansouni

**Affiliations:** McGill University Health Centre, Montreal, Quebec, Canada (L.B. Harrison, M.D. Libman, C. Caya, C.P. Yansouni);; National Reference Centre for Parasitology, Montreal (M. Ndao)

**Keywords:** Trichinella, trichinellosis, eosinophilia, parasites, laboratory, hematologic, biochemical, Quebec, Canada

## Abstract

Prolonged eosinophilia is characteristic of trichinellosis. To determine the optimal eosinophil threshold for reflex *Trichinella* testing, we examined all 43 cases in Nunavik, Quebec, Canada, during 2009–2019. Using receiver operating characteristic analysis, we determined that eosinophil counts >0.8 × 10^9^ cells/L should prompt consideration of trichinellosis and testing to rapidly identify potential outbreaks.

*Trichinella nativa* infection is associated with ingestion of parasitized sylvatic animals and periodic outbreaks among residents of northern Canada (*1*–*3*). In the Arctic region of Nunavik in Quebec, outbreaks associated with polar bear and walrus consumption have prompted public health interventions, including a highly successful community-led active surveillance system that examines hunted meat for evidence of *Trichinella* encystment (*4*,*5*). We report a 10-year case series of *Trichinella* infection in Nunavik and describe the laboratory features. Eosinophilia is a well-characterized feature of infection that is readily available for most cases. We performed receiver operating characteristic (ROC) analysis to define an optimal threshold of eosinophilia to prompt reflex *Trichinella* antibody testing and rapid reporting to public health authorities for timely outbreak investigation (*1*–*3*).

In a retrospective test-negative case–control study, we reviewed laboratory and public health records to identify cases of trichinellosis in Nunavik that occurred from 2009 through 2019. Our study was approved by the Research Institute of the McGill University Health Centre Research and Ethics Board (REB #2020-5312). 

We first reviewed all requests for *Trichinella* serologic testing sent from Quebec to the National Reference Centre for Parasitology, the only testing site for Quebec, during 2009–2019 (Appendix). To define an initial set of cases (with positive *Trichinella* serologic results), we selected specimens originating from Nunavik. One author (L.B.H.) reviewed the charts and confirmed cases if the clinical evolution was compatible with the positive serologic results. Because trichinellosis is notifiable by provincial law, we cross-referenced cases with the public health database to identify other cases determined epidemiologically and reviewed those charts. We defined a set of region-matched controls as those with negative *Trichinella* serologic results. Those controls are therefore persons from the general population from the same region who had clinical manifestations that prompted testing for trichinellosis. Although serologic results early in the disease course could be negative, chart review of controls did not yield additional suspected cases on the basis of clinical evolution. We extracted available clinical and laboratory data by chart review at the McGill University Health Centre and at regional health centers in Nunavik. We calculated summary statistics and tests (*t*-test and χ^2^), comparing cases and controls by using R (*6*), and generated ROC curves by using the pROC R package (*7*).

We identified 43 cases of trichinellosis and a set of 31 region-matched controls (Table). We excluded 4 possible case-patients with weakly positive serologic results but ambiguous clinical manifestations consistent with past infection. Information on signs and symptoms was available for only 19/43 case-patients, but demographic, laboratory, and clinical outcomes were well documented. 

**Table:** Characteristics of patients and controls in study of *Trichinella* infections in Nunavik, Quebec, Canada, 2009–2019*

Characteristic	Case-patients, n = 43	Controls, n = 31	Test statistic†	p value
Demographics				
Age, y	39.1 (5–75, 16.1)	45.8 (0–80, 22.4)	*t* = –1.48 (–15.59 to 2.32)	0.144
Female	30 (69.8)	16 (51.6)	χ^2^ = 3.77	0.052
Male	13 (30.2)	15 (48.4)		
Level of care received, no. with available information/total no. (%)				
Outpatient	18/27 (67)	15/24 (63)	NA	NA
Inpatient	9/27 (33)	9/24 (38)	NA	NA
Critical care	0/27	0/24	NA	NA
Evacuated to southern Quebec	7/27 (26)	8/24 (33)	NA	NA
Unknown	16/43 (37)	7/31 (23)	NA	NA
Positive *Trichinella* serologic result	39 (91)§	NA	NA	NA
Biochemical features during illness				
Eosinophils, × 10^9^ cells/L	5.35 (0.80–17.40, 3.81)	0.80 (0–4.5, 1.00)	*t* = 6.47 (3.14 to 5.95)	<0.001
Platelets, × 10^9^/L	545 (294–977, 169)	479 (208–1009, 210)	*t* = 1.45 (–24.43 to 154.90)	0.151
Creatinine kinase, U/L	1562 (103–8081, 1511)	956 (28–6470, 1730)	*t* = 1.42 (–245.92 to 1,458.90)	0.160
C-reactive protein, mg/L	66.5 (8–191.7, 42.1)	66.6 (0.5–253.0, 82.9)	*t* = –0.03 (–38.05 to 36.83)	0.974
Alanine aminotransferase, U/L	140.2 (21.0–541.0, 128.1)	85.1 (16.0–334.0, 75.6)	*t* = 1.82 (–5.56 to 115.91)	0.074

*Values are no. (%) or mean (range, SD) except as indicated. NA, not applicable; *t*, Student *t *statistic. †Expressed as *t* value (95% CI). ‡Optical density >0.3. §Four cases were determined epidemiologically, and initially negative serologic testing was not repeated.

Case-patients had a median age of 40 years and were mostly female (30/43, 69.8%), which may result from chance (p = 0.052), differential exposure to parasitized meat, food sharing, or food preparation practices; or selection bias (*8*). When available, clinical features were similar to those previously described for trichinellosis (i.e., fever, rash and myalgia) (*9*). No patients died, and 9/27 (33%) patients with documented illness required hospitalization. Epidemiologic investigations revealed sporadic cases and 2 suspected point-source outbreaks (*8*). In 1 outbreak, seals were suspected as the source of infection, which could represent a change in epidemiology from previous outbreaks associated with polar bear and walrus meat and might reflect the surveillance program targeting game meat from the latter animals but not seals.

Laboratory information was available for 41/43 case-patients, a larger series of findings in *Trichinella* infection in Nunavik than previously reported. Features of *Trichinella* infection in Nunavik, presumptively caused by *T.* *nativa*, are similar to those reported for *T. spiralis* infection (*9*), including elevated creatinine kinase and eosinophilia (Table). The variable that differed most between cases and controls was peak absolute eosinophilia (5.35 vs. 0.80 × 10^9^ cells/L; p<0.001). Among case-patients, peak eosinophilia was noted early and declined over months; among controls, counts were frequently elevated but stable over time (Appendix Figure). Using ROC analysis, we identified an absolute eosinophilia threshold of >0.8 × 10^9^ cells/L, which identified all cases in this series with a specificity of 71% (Figure). We assessed the potential effect on resource use of this threshold by examining the region’s whole-population distribution of absolute eosinophil counts. Among 8,562 persons who submitted a specimen for complete blood count for any reason from January 2019 through April 2022, a total of 287 had eosinophil counts that exceeded our threshold (86 [1.2%] specimens/year).

**Figure:**
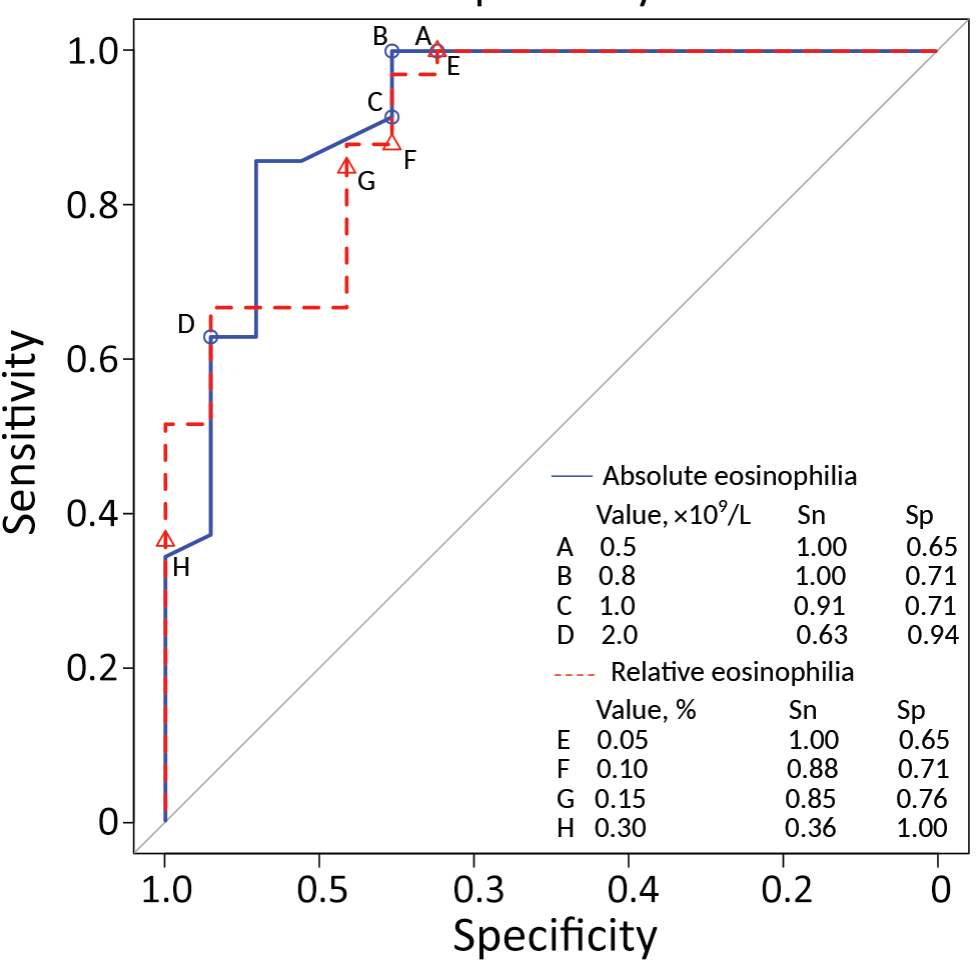
Receiver operating characteristic curve comparing performance of thresholds of absolute and relative eosinophilia to trigger automatic reporting of possible trichinellosis. Sn and Sp for the thresholds of absolute eosinophilia examined were 0.5 × 10^9^ (Sn = 1.0, Sp = 0.65), 0.8 × 10^9^ (Sn = 1.0, Sp = 0.71), 1.0 × 10^9^ (Sn = 0.91, Sp = 0.71), 2.0 × 10^9^ (Sn = 0.63, Sp = 0.94) and for relative eosinophilia were 5% (Sn = 1.0, Sp = 0.65), 10% (Sn = 0.88, Sp = 0.71), 15% (Sn = 0.85, Sp = 0.76), and 30% (Sn = 0.36, Sp = 1.0). Sn, sensitivity; Sp, specificity.

Automated flags and reflex testing in Nunavik now incorporate the threshold identified in our analysis. In the absence of a defined alternative diagnosis, eosinophil counts of >0.80 ×10^9^ cells/L should prompt clinical consideration of trichinellosis and further investigation. Early identification of outbreaks is critical in this region—where hunted meat is shared widely within and among communities—to limit exposures and enable delivery of postexposure prophylactic anthelmintic therapy, which has evidence of effectiveness in this serious illness (*1*,*4*,*10*). The cost–benefit ratio of this threshold will require ongoing assessment.

Appendix Supplemental information for study of eosinophilia threshold for *Trichinella* testing, Nunavik, Quebec, Canada, 2009–2019.
