# Correction: Vol. 26, No. 12

**DOI:** 10.3201/eid2905.C12905

**Published:** 2023-05

**Keywords:** errata, erratum, correction

The Figure had an incorrect x-axis scale in Laboratory Features of Trichinellosis and Eosinophilia Threshold for Testing, Nunavik, Quebec, Canada, 2009–2019 (L.B. Harrison et al.). The article has been corrected online (https://wwwnc.cdc.gov/eid/article/28/12/22-1144_article).

