# Supplementary material for: Laboratory Features of Trichinellosis and Eosinophilia Threshold for Testing, Nunavik, Quebec, Canada, 2009–2019
Source: Emerg Infect Dis. 2022 Dec;28(12):2567–9. doi: 10.3201/eid2812.221144 (PMC9707581; doi:10.3201/eid2812.221144)
Supplement: Appendix — Supplemental information for study of eosinophilia threshold for Trichinella testing, Nunavik, Quebec, Canada, 2009–2019. [file 22-1144-Techapp-s1.pdf]

Article DOI: https://doi.org/10.3201/eid2812.221144

Laboratory Features of Trichinellosis and Eosinophilia Threshold for Testing, Nunavik, Quebec, Canada, 2009–2019

Appendix

Trichinella serology testing at the Canadian National Reference Centre for Parasitology (NRCP)

Trichinella serology offered by the NRCP detects host antibodies against *Trichinella* species. The serological assay is a non-commercial laboratory-developed indirect enzyme-linked immunosorbent assay (ELISA). A crude antigen preparation derived from *Trichinella spiralis* L3-stage larvae is used. This antigen preparation shares common epitopes with other *Trichinella* species and cross-reacts. The assay is optical density based and a serology is considered positive at an OD > 0.35. The assay has been performed in the same manner over the study period of this case series (2009-2019).

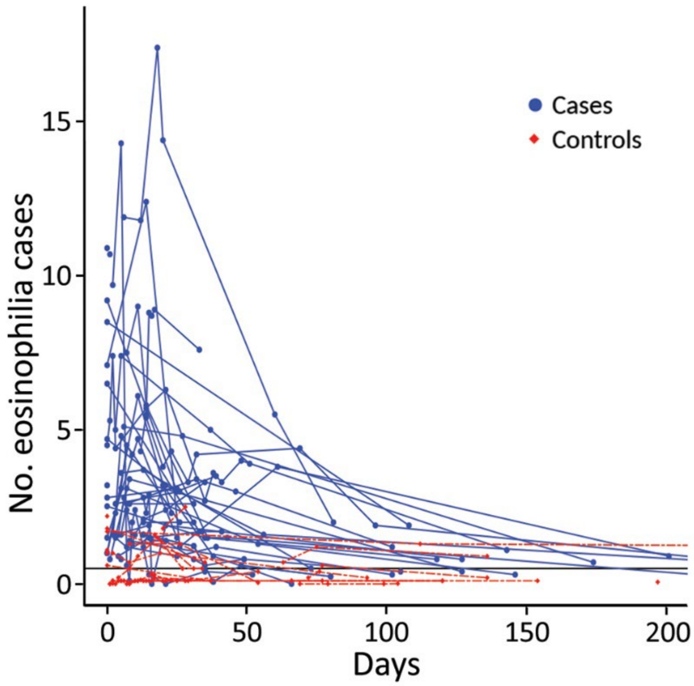

Appendix Figure. Time course of absolute eosinophilia for case-patients (blue dots, solid lines) and controls (red diamonds, dashed line) since presentation for medical care for acute *Trichinella* infection, Arctic Region of Nunavik, Quebec, Canada, 2009–2019. Points are joined for individual patients when >1 measurement was available. The horizontal line at 0.45×10^9 represents the upper limit of normal for eosinophilia in adults at McGill University Health Centre.
